# Inebriety and the So-Called Cures

**Published:** 1905-06

**Authors:** James Stewart

**Affiliations:** Resident Physician of the Dunmurry Home for Inebriate Gentlefolk


### INEBRIETY AND THE SO-CALLED CURES.

BY

JAMES STEWART, B.A., F.R.C.P. Edin.,

*Resident Physician of the Dunmurry Home for Inebriate Gentlefolk.*

---

INACCURACY lies at the root of many failures. The physician who is inaccurate in the use of terms connected with disease incurs a great responsibility. Medical practitioners are much to blame for the frequency with which the terms "drunkenness" and "inebriety" are confounded the one with the other.

The pathologist knows that a man may be a drunkard for years and years and yet never become an inebriate. (Inebriety is an organic as well as a functional disease: it may, shortly, be defined as "a disease of the brain which has gone so far as to affect the will-power.") On the other hand, a man may suffer from the disease of inebriety although he never was "drunk" in his life. It cannot be too often repeated that "inebriety" and "drunkenness" are not convertible terms. Medical men should take every opportunity of pointing out this difference. If they did so, the Yankee quacks, the English miracle-workers, and other charlatans who profess to cure inebriety in a few weeks would soon disappear from the land.

If, however, the profession fails to point out this distinction, then police magistrates, journalists, clergymen, and others who are considered to be authorities on correct diction will continue to do the positive injury that results from the two words being used indiscriminately.

The injury is twofold. In the first place, it suggests to the drunkard an excuse for his conduct which has no scientific basis, and causes sympathy to be excited for him which is entirely misplaced. Secondly, the quack gets credit for curing a physical disease, whereas he has merely cast a spell, so to speak, over the drunkard, and got him to give up his debauch for a while. He gets time, as it were, to reflect on the injury he is doing to his own health, to his business, and to his professional or other prospects, and also on the distress he is causing to those near and dear to him. In a few cases the pause thus given enables the drunkard to "pull himself together." If he does *not* return to his debauch the quack flaunts the case as one of "inebriety" cured, he says, by the injection of gold, or what not!

The canon, the philanthropic countess, *et hoc genus omne*, are quickly told of the wonderful recovery. They are asked to form themselves into a committee of investigation, and the patient is invited to present himself before these good folk periodically and relate his experiences. He does so, and each time assures his examiners that he has never touched a drop of intoxicating drink since the "cure" was completed by "Dr. Topsy" or whoever the quack may have been. He relates how he had found it impossible previously to pass a public-house without going in for a drink, whereas since he left "Dr. Topsy's" home he has never had any difficulty of the kind.

Forthwith the lightning curer—"Dr. Topsy" we'll call him—says to the committee: "You see, gentlemen, the disease in this case has been thoroughly and permanently cured by my system. Allow me to quote you in my prospectus as satisfied of its efficiency." The prospectus appears shortly with the names of distinguished philanthropists and so on. But among all the names there is none of any physician of repute.

A few months go by, during which hundreds of pounds are spent by the Yankee syndicate in advertising the "Topsy Treatment" and its marvellous success. Then comes a notice in the column of the daily paper of a company being on the point of formation to work this "discovery" on an extended scale, and offering exceptional advantages to foundation shareholders in the medical profession.

Far be it from me to impugn the *bona fides* of these supporters of the nostrum-monger. My contention is that it is through ignorance that they have been led into error. They have not the knowledge possessed by the trained medical man. They have confounded two different things—a disease and a vice. Inebriety is a disease, drunkenness is a vice. The latter may, and often does, bring on the disease; but inebriety may become an established lesion without the patient having ever been a "drunkard" in the ordinary acceptation of the term.

The following statements, founded on observations extended over many years of clinical work, I would humbly suggest might with advantage be put before an intelligent inquirer into the value of these so-called "cures" of inebriety:—

(1) The nervous system varies considerably in different individuals. In some cases the brain is much more susceptible to the poisonous effects of alcohol than in others. For example, the neurotic will be affected prejudicially by a comparatively small quantity of spirits which might be taken—aye, even double as much—with almost entire impunity by his neighbour of a different type.

(2) Alcohol has an especial affinity for the "memory centre" and the "will centre" of the brain. When either or both of these become deranged—but not till then—the case may be pronounced to be one of inebriety.

(3) In the inebriate the cells of these centres are physically injured. An organic lesion has been established. Till other cells have been requisitioned, so to speak, to take the place of those that have been deranged, the functions previously exercised through the latter must remain in abeyance.

(4) The process is a very slow one—this taking up of new work by a set of cells not previously so employed. It cannot be hurried. Gold will not expedite the process, nor apomorphine, nor any drug with which we are acquainted. The restoration of healthy function has never been known by the writer during an experience of over twenty years at Dunmurry to be accomplished in a single case in less than a twelvemonth. An official document issued by the Home Secretary in 1899 stated that there was a consensus of opinion among medical men that, in order to give a chance of effective operation to even the best designed method of treatment, a considerable period of residence in a "home" was required. The treatment "cannot be successfully carried through under eighteen months to two years even in favourable cases."

(5) Hypnotism, the injection of apomorphine or other drug

(according to "Dr. Topsy's" or any other method) may arrest the drunkard in his downward course of vice. It will not do anything towards the re-building of injured brain tissue.

(6) The inebriate must for at least twelve months be placed in such surroundings as experience shows are favourable for the carrying out of the work of restoration of the injured brain cells—a work which must not be interrupted, and which cannot go on if alcohol in any quantity, even the smallest, be taken by the patient. The protection of the patient can only be secured in a properly organised "home."

(7) Forty-one per cent. of the cases of both sexes treated at the Dunmurry Home during the last ten years had previously been under the care of "Dr. Topsy" or some other lightning curer of inebriety. In every one of these cases without exception the patient said the benefit was only temporary, the duration of the immunity depending on (*a*) the occupation or surroundings of the patient after the so-called "cure" had been effected and (*b*) the condition of the general health. All looked upon the expenditure on the "cure" as waste of both time and money.

In conclusion, I would like to ask this question: If my experience be such as is put forward in the last paragraph, can one be surprised at so many medical men having no faith in any treatment of the disease of inebriety? Legitimate practitioners who devote their whole time and energy to the treatment and supervision of a few cases suffer because (1) work is undertaken by those unable to perform the task—no general practitioner, for instance, can exercise the necessary constant *personal* supervision; and (2) money has been spent on the quacks which would have gone towards the payment of charges in a properly-constituted "home," the friends being thus proportionately less able to make a second attempt on behalf of the inebriate.

